# Role of the TLR4 pathway in blood-spinal cord barrier dysfunction during the bimodal stage after ischemia/reperfusion injury in rats

**DOI:** 10.1186/1742-2094-11-62

**Published:** 2014-03-28

**Authors:** Xiao-Qian Li, Huang-Wei Lv, Wen-Fei Tan, Bo Fang, He Wang, Hong Ma

**Affiliations:** 1 Department of Anesthesiology, First Affiliated Hospital, China Medical University, Shenyang 110001, Liaoning, China

**Keywords:** Blood spinal cord barrier, Myeloid differentiation factor 88, Spinal cord ischemia-reperfusion injury, TIR domain-containing adaptor-inducing IFN-β, Toll-like receptors 4

## Abstract

**Background:**

Spinal cord ischemia-reperfusion (I/R) involves two-phase injury, including an initial acute ischemic insult and subsequent inflammatory reperfusion injury, resulting in blood-spinal cord barrier (BSCB) dysfunction involving the TLR_4_ pathway. However, the correlation between TLR_4_/MyD_88_-dependent and TLR_4_/TRIF-dependent pathways in BSCB dysfunction is not fully understood. The aim of this study is to characterize inflammatory responses in spinal cord I/R and the events that define its clinical progression with delayed neurological deficits, supporting a bimodal mechanism of injury.

**Methods:**

Rats were intrathecally pretreated with TAK-242, MyD_88_ inhibitory peptide, or Resveratrol at a 12 h interval for 3 days before undergoing 14-minute occlusion of aortic arch. Evan’s Blue (EB) extravasation and water content were detected at 6, 12, 18, 24, 36, 48, and 72 h after reperfusion. EB extravasation, water content, and NF-κB activation were increased with time after reperfusion, suggesting a bimodal distribution, as maximal increasing were detected at both 12 and 48 h after reperfusion. The changes were directly proportional to TLR_4_ levels determined by Western blot. Double-labeled immunohistochemical analysis was also used to detect the relationship between different cell types of BSCB with TLR_4_. Furthermore, NF-κB and IL-1β were analyzed at 12 and 48 h to identify the correlation between MyD_88_-dependent and TRIF-dependent pathways.

**Results:**

Rats without functional TLR_4_ and MyD_88_ attenuated BSCB leakage and inflammatory responses at 12 h, suggesting the ischemic event was largely mediated by MyD_88_-dependent pathway. Similar protective effects observed in rats with depleted TLR_4_, MyD_88_, and TRIF receptor at 48 h infer that the ongoing inflammation which occurred in late phase was mainly initiated by TRIF-dependent pathway and such inflammatory response could be further amplified by MyD_88_-dependent pathway. Additionally, microglia appeared to play a major role in early phase of inflammation after I/R injury, while in late responding phase both microglia and astrocytes were necessary.

**Conclusions:**

These findings indicate the relevance of TLR_4_/MyD_88_-dependent and TLR_4_/TRIF-dependent pathways in bimodal phases of inflammatory responses after I/R injury, corresponding with the clinical progression of injury and delayed onset of symptoms. The clinical usage of TLR_4_ signaling inhibitors at different phases may be a therapeutic option for the prevention of delayed injury.

## Introduction

Spinal cord ischemic/reperfusion (I/R) injury is a serious complication of thoracoabdominal aortic surgery. The injury occurs as a two-phase process that correlates with its onset and delayed clinical progression. The first phase includes metabolic and inflammatory processes following ischemia, and the second one is a set of amplified inflammatory responses mediated by biochemical cytokines after restoration of spinal cord blood flow [1,2]. Clinically, this is observed as the bimodal distribution of paraplegia, in which lower limb movement is observed immediately after operation but later deteriorates [3-5]. This phenomenon was reported to occur in 2% to 18% of patients, and a total of 50% of patients displayed delayed neurological deficits due to multifactorial causes [5]. The blood-spinal cord barrier (BSCB), consisting of continuous capillary endothelial cells surrounded by astrocytes and pericytes as well as perivascular microglia, is critical to maintain spinal cord homeostasis and neurologic function [6,7]. Impairment of the BSCB may induce spinal cord edema and progressive breakdown of BSCB integrity, which may aggravate injury, resulting in paraplegia or even death, thus highlighting the complexity of the injury [1,3,8,9]. Previous studies have demonstrated that Toll-like receptors 4 (TLR_4_), a class of transmembrane proteins that can recognize specific ligands extracellularly, are closely associated with the inflammation that occurs after I/R injury and mediate the pathogenesis of hind-limb function and neuronal viability [10-12]. The myeloid differentiation factor 88 (MyD_88_) and TIR domain-containing adaptor-inducing IFN-β (TRIF), which function via the up regulation of TLR_4_, are the two most important adaptor proteins that provoke the transduction of common downstream signaling molecules, such as NF-κB, into the nucleus and regulate the expression of target inflammatory genes [13-15]. In the MyD_88_-dependent pathway, previous studies have demonstrated that TLR_4_ directly activates MyD_88_ and thus facilitates further activation of NF-kB and downstream inflammatory cytokine interleukin (IL)-1β in the early phase [10,13,16]. However, recent studies suggest that TLR_3_ is not indispensable for the TRIF-dependent pathway [17]. In the TLR_4_ signaling pathway, TRIF is recruited upon receiving stimuli from TICAM-2, leading to rapid activation of interferon regulatory factor 3 and beta interferon, which in turn activates TRIF and MyD_88_[15-17] and induces delayed NF-kB activation [18]. In our previous studies, we have reported the breakdown of BSCB integrity in the spinal cord after I/R injury [8,9]. Therefore, procedures that can preserve the intact function of the BSCB and attenuate the inflammatory responses after I/R injury would improve prognosis. However, not much is known about the underlying roles of TLR_4_ signaling and the transduction of its downstream adaptor receptors. The aim of this study is to investigate the role of TLR_4_ in increased BSCB leakage using a specific cell population during a post-injury bimodal stage. We established a functional deletion of TLR_4_, MyD_88_, and TRIF receptor by intrathecal treatment with TAK-242 [19], MyD_88_ inhibitory peptide (MIP) [16], or Resveratrol [20] to prevent TLR_4_ from interacting with its downstream receptors, namely, MyD_88_ and TRIF. In addition, we explored the underlying relevance of TLR_4_/MyD_88_-dependent and TLR_4_/TRIF-dependent pathways in the bimodal phase of inflammatory responses after I/R injury by determining the activation of NF-kB and subsequent products of the proinflammatory cytokine, IL-1β.

## Materials and methods

### Experimental animals

All experimental procedures were approved by the Ethics Committee of China Medical University and were performed in accordance with the *Guide for the Care and Use of Laboratory Animals* (U.S. National Institutes of Health publication No. 85–23, National Academy Press, Washington DC, revised 1996). The rats used in this study were all male Sprague-Dawley rats weighing between 200 and 250 g, neurologically intact before anesthesia, and expressed normal, functional TLR_4_. The rats were bred in standard cages with free access to food and water, and were housed separately after surgery at the First Affiliated Hospital, China Medical University.

### Animal surgical procedures

The spinal cord I/R model was induced by occluding the aortic arch for 14 min, as previously reported [21]. In general, all rats were anesthetized with an intraperitoneal injection of 4% sodium pentobarbital at an initial dose of 50 mg/kg. The aortic arch was exposed through a cervicothoracic approach. Under direct visualization, the aortic arch was cross-clamped between the left common carotid artery and the left subclavian artery. A laser Doppler blood flow monitor (Moor Instruments, Axminster, Devon, UK) was used to confirm complete occlusion. Ischemia was confirmed as a 90% decrease in flow measured at the femoral artery that continued for 14 min, after which the clamps were removed and followed by 72 h of reperfusion. Sham operation rats underwent the same procedure, but no occlusion of the aortic arch was performed.

### Experimental protocol

Rats were randomly assigned to one of the five groups: Sham, Ischemia/reperfusion (I/R), MIP, Resveratrol, and TAK-242 (TAK) group. Continuous intrathecal injections were performed at 12 h intervals for 3 days prior to ischemia or the induction of sham surgery. In order to study the role of TLR_4_-mediated signal pathways in I/R injury, the rats were intrathecally injected with 10 μL MIP (50 nmol/μL), Resveratrol (50 nmol/μL), TAK-242 (10 nmol/μL), or an equivalent volume of normal saline as control at L_4–6_ segments of the spinal cord, as previously described [16,19,20].

### Examination of blood-spinal cord barrier leakage

Measuring the water content of the spinal cord and Evan’s Blue (EB) extravasation were, respectively, the most common methods used for the quantitative and qualitative examination of BSCB leakage after spinal cord I/R injury. The percentage of water content was calculated as: (wet weight - dry weight)/wet weight × 100, using a wet-dry method.

For quantification of EB extravasation, 30 g/L EB (45 mg/kg; Sigma, St. Louis, MO, USA) was slowly administered intravenously in the tail vein 60 minutes before sacrificing the animals. After soaking the tissues in methanamide for 24 h (60°C) and centrifugation at 20,000 × *g* for 20 min, the absorption of the supernatant was detected at 632 nm and reported as the amount of EB per wet tissue weight (μg/g). For dye fluorescence measurements, the tissue was fixed in 4% paraformaldehyde, then sectioned (10 μm) and visualized using a BX-60 (Olympus, Melville, NY, USA) fluorescence microscope (green zone).

### Immunofluorescence staining

Double immunofluorescence analysis was performed to measure the activation and localization of TLR_4_ after I/R injury. The spinal cord was fixed and sectioned into 10-μm slices with a Leica CM3050 S cryostat. The sections were blocked with 10% bovine serum albumin for 1 h at room temperature and incubated overnight at 4°C with the primary antibodies: mouse anti-TLR_4_ (1:100, Abcam, Cambridge, UK), rabbit anti-CD31 antibody (1:800, Abcam), rabbit anti-CD13 antibody (1:500, Abcam), rabbit anti-Iba-1 antibody (1:800, Wako, Germany), and rabbit anti-GFAP antibody (1:800, Abcam). After incubation with Alexa 594-conjugated donkey anti-mouse IgG (1:500, Molecular Probes, Eugene, Oregon, USA) and Alexa 488-conjugated donkey anti-rabbit IgG (1:500, Molecular Probes) for 2 h at room temperature, the images were captured using a Leica TCS SP2 (Leica Microsystems, Buffalo Grove, IL, USA) laser scanning microscope and photographed by the attached digital camera to determine the number of immunoreactive cells. Non-specific staining was determined by omitting the primary antibody. The data were expressed as numbers of positive cells/area/spinal section ± standard error mean (SEM).

### Biochemical analysis

The spinal cord was collected, homogenized, and centrifuged. The level of IL-1β was determined by ELISA kit (R & D Systems, Minneapolis, MN, USA). According to the manufacturer’s instructions, the absorbance was detected at 450 nm and the content of each sample was calculated from the standard curve and the quantity of IL-1β was expressed as pg/mg protein.

### Western blot analysis

The expression of TLR_4_, MyD_88_, TRIF, and NF-κB p65 in spinal cord tissue were determined by Western blot. The rats’ spinal cords were homogenized, and the total proteins were purified using cell and tissue protein extraction reagents according to manufacturer’s instructions (KC-415; KangChen, Shanghai, China). The antibodies used in this experiment were mouse monoclonal anti-TLR_4_ (1:500, Abcam), rabbit polyclonal anti-MyD_88_ (1:500, Abcam), rabbit polyclonal anti-TRIF (1:500, Abcam), and rabbit polyclonal anti- NF-κB p65 (1:500, Abcam), along with horseradish peroxidase conjugated secondary antibodies (Bioss, Beijing, China). Semi-quantitation of scanned images was performed using Quantity One software (Bio-Rad Laboratories, Milan, Italy).

### Statistical analysis

All data were expressed as means ± standard error mean (means ± SEM) and analyzed by SPSS software (version 17.0, SPSS Inc., Chicago, IL, USA). All variables measured in this study were normally distributed, and the groups were compared with Student’s *t*-test or one-way analysis of variance (ANOVA), followed by Newman-Keuls post-hoc analysis. A *P* value of < 0.05 was considered statistically significant.

## Results

### Effects of BSCB leakage after I/R

**Figure 1:**
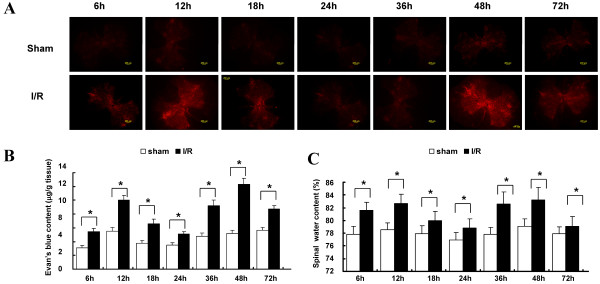
**Time dependent progression of BSCB permeability changes after spinal cord ischemia/reperfusion (I/R) injury. (A)** Effects on Evans blue (EB) extravasation in the Sham and I/R groups along the entire time course from 6 to 72 h after surgery. I/R-induced a significant increase in EB extravasations at each observed time point, with the greatest intensity at 12 and 48 h, especially in the gray matter. Scale bars = 200 μm. **(B)** Quantification of EB content of the spinal cord (μg/g). **(C)**  Quantification of the water content of the spinal cord. All data are represented as means ± SEM (n = 8 per group). *P < 0.01 vs. Sham group.

To determine the time frame of BSCB leakage and explore the underlying mechanisms, we performed a time course of I/R injury from 6 to 72 h post-operation determined by the extravasation of EB dye, which was visualized as red fluorescence under the fluorescent microscope (Figure 1A). Since the L_4–6_ segments of the spinal cord are most vulnerable to ischemic injury [22], the increased BSCB leakage was analyzed at this site. Our observations suggest that the maximal differences in EB extravasations between the Sham group and I/R group occurred at 12 and 48 h after surgery. Quantification of EB content in the injured spinal cord revealed that it reached maximal EB extravasations in the I/R group at 12 h, followed by delayed and aggravated BSCB leakage beginning at 36 h, peaking again at 48 h (Figure 1B). Similarly, differences in water content attributed to the increased BSCB leakage in the I/R group also had a bimodal distribution, peaking at 12 and 48 h (Figure 1C). There were significant differences between the two groups at all observed time points (*P* < 0.05 vs. Sham group).

### Effects of TLR_4_ immunoreactivity after I/R

**Figure 2:**
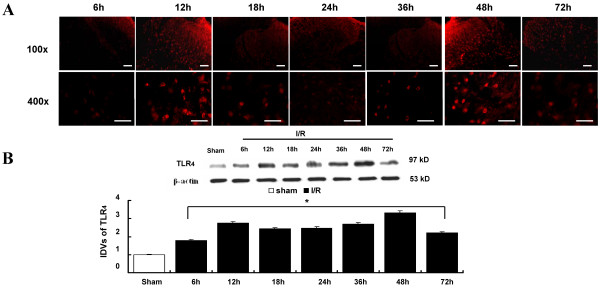
**Time course of TLR**_**4 **_**activation in the spine after I/R injury. (A)** Representative immunofluorescence of TLR_4._ Prominent TLR_4_ immunoreactivation was observed in both dorsal horns of the spinal cord in operated rats. Spinal cord I/R-induced TLR_4_-up regulation increased with time and peaked at 12 and 48 h after surgery. Scale bars = 100 μm for the 100× and 50 μm for the 400× images. **(B)** Representative Western blot analysis and integrated density values (IDVs) of TLR_4_ activation. The IDVs of TLR_4_ in the I/R group with different reperfusion time points were calculated after normalizing against the Sham group and presented as relative protein expression units. The maximal of IDVs were observed at 12 and 48 h after surgery. All data are represented as means ± SEM (n = 8 per group). *P < 0.05 vs. Sham group.

Immunofluorescence and Western blot analyses for TLR_4_ performed during the time course of I/R injury were shown in Figure 2A. The expression of TLR_4_ significantly increased with time in spinal cords of the I/R group, where the TLR_4_ protein was expressed at maximum levels at 12 and 48 h after surgery.

### Blocking effects of the TLR_4_ pathway after I/R

**Figure 3:**
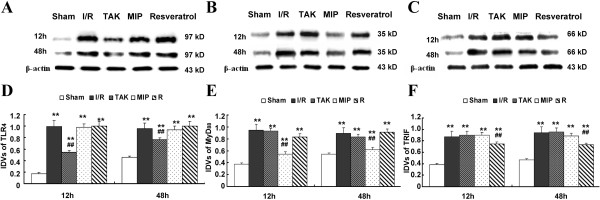
**Blocking effects of specific binding protein on TLR**_**4**_**, MyD**_**88**_**, and TRIF protein. (A-C)** Representative Western blot analysis of TLR_4_, MyD_88_, and TRIF protein in L_4–6_ segments of spinal cord at 12 and 48 h after I/R injury. **(D-F)** The integrated density values (IDVs) in each group with different reperfusion time points were calculated after normalizing against β-actin and presented as relative protein expression units. Data are presented as means ± SEM (n = 8 per group). **P < 0.01 vs. Sham group; ## *P* < 0.01 vs. I/R group.

We established a functionally compromised TLR_4_, MyD_88,_ and TRIF by intrathecal pretreatment with TAK-242, MIP, and Resveratrol, and analyzed the blocking effects of each group at 12 and 48 h after surgery since BSCB leakage was maximal at these two time points. As the cross-talk in signal transduction used to verify the specification and blocking effects of the inhibitors, the changes in protein level of TLR_4_, MyD_88_, and TRIF were detected by Western blot (Figure 3A-C). Quantitative Analysis showed that I/R injury induced TLR_4_, MyD_88_, and TRIF expressions in all groups compared to the Sham group at 12 and 48 h after injury (Figure 3 D-F, all *P* < 0.05 vs. Sham group). Pretreatment with TAK-242 effectively prevented the up regulation of TLR_4_ without much influence on expressions of MyD_88_ and TRIF, suggesting satisfactory specificity and effectiveness of TAK-242 (Figure 3A and D, *P* < 0.05). Likewise, following targeted down regulation of MyD_88_ and TRIF by intrathecal pretreatment with MIP and Resveratrol, respectively, there were no significant differences in expressions of the other two indicators (TLR_4_ and TRIF or TLR_4_ and MyD_88_) (Figure 3B and E *vs* C and F, both *P* > 0.05).

### Effects of the TLR_4_ pathway on colocalization of different cell types in blood spinal cord barrier after I/R

**Figure 4:**
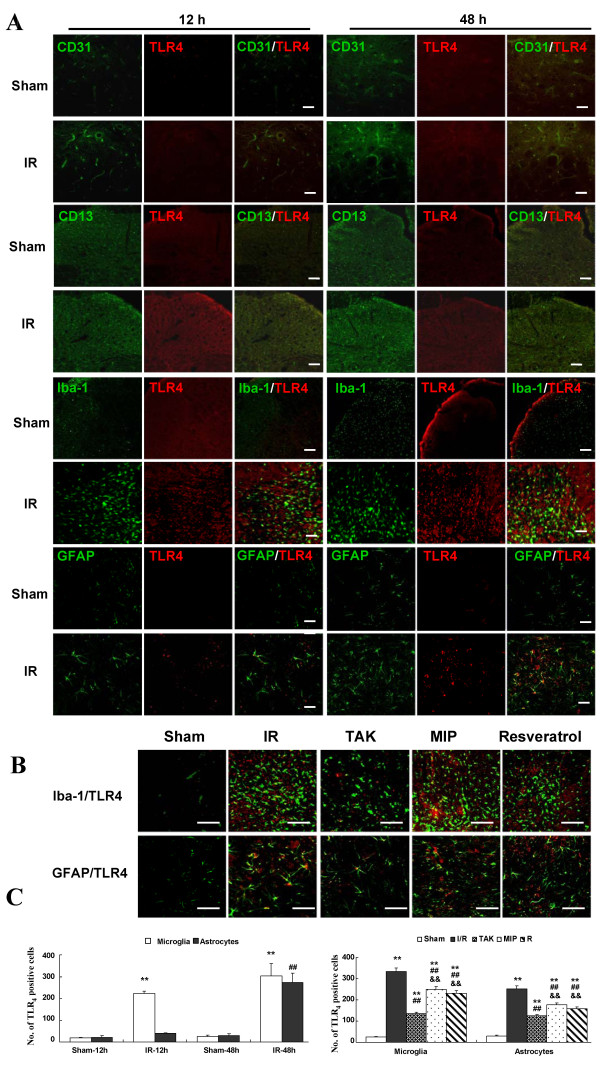
**Double-labeled immunofluorescence TLR**_**4 **_**receptor and specific cell population in blood spinal cord barrier after spinal cord I/R injury. (A)** Representative immunohistochemical localization of capillary endothelial cells (CD31; green), pericytes (CD13; green), microglia (Iba-1; green), astrocytes (GFAP; green), and Toll-like receptor (TLR_4_; red) in spinal cords of sham-operated or operated rats at 12 and 48 h after the surgical procedure, respectively. Scale bars = 100 μm for the 200× images. **(B)** Effects of TLR_4_ signaling on TLR4 co-localization cells. Representative double-labeling micrographs show that intrathecal pretreatment with TAK-242, MIP, and Resveratrol before I/R injury significantly prevented microglia and astrocytes upregulated expression of TLR_4_ at 48 h after the surgery_._ Scale bars = 50 μm for the 400× images. **(C)** Quantification of TLR_4_-positive microglia and TLR_4_-positive astrocytes in the spinal cords. Data are presented as mean ± SEM (n = 6). **P < 0.01 vs. Sham group; ## *P* < 0.01 vs. I/R group; &&*P* < 0.05 vs. TAK group.

Continuous capillary endothelial cells, pericytes, astrocytes, and perivascular microglia are the major cellular components of the BSCB. With the understanding that the TLR_4_ pathway may play an important role in the inflammatory damage to BSCB integrity, we further identified a specific cell population in BSCB with the following cellular markers: CD31 (platelet endothelial cell adhesion molecule-1, capillary endothelial cell marker), CD13 (aminopeptidase-N, pericyte marker), Iba-1 (microglial marker), and GFAP (astrocyte marker) (Figure 4). Based on our results, the majority of TLR_4_ was co localized with the distribution of Iba-1-positive microglia in rats of the I/R group at both 12 and 48 h after surgery, but not in sham-operated ones, confirming that TLR_4_ was indeed upregulated in microglia. Similarly, the increasing numbers of double-labeled astrocytes were found in rats of the I/R group at 48 h after surgery, suggesting the involvement of astrocytes. However, no identical fluorescence label of TLR_4_ was found in capillary endothelial cells and pericytes of rats undergoing ischemia or sham-operation at the above time points. Quantification of TLR_4_ co-localization in Figure 4C confirmed that the majority of TLR_4_ was expressed in microglia at 12 h after injury, while at 48 h after surgery, TLR_4_was comparably expressed in both microglia and astrocytes (all *P* < 0.01 vs. Sham group).

Additionally, we determined the effects of the TLR_4_ pathway on double-labeled microglia and astrocytes at 48 h after I/R injury for the most double-labeled cells observed at that time. As shown in Figure 4B, the maximal number of double-labeled microglia and astrocytes were both observed in the I/R group. Compared with I/R group, the number of double-labeled microglia and astrocytes were decreased the most in the TAK group and the least in the MIP group (*P* < 0.05 vs. I/R group, *P* > 0.05 vs. MIP group). Almost no double-labeled glial cells were detected in the Sham group at the above time point.

### Effects of the TLR_4_ pathway on BSCB leakage after I/R

**Figure 5:**
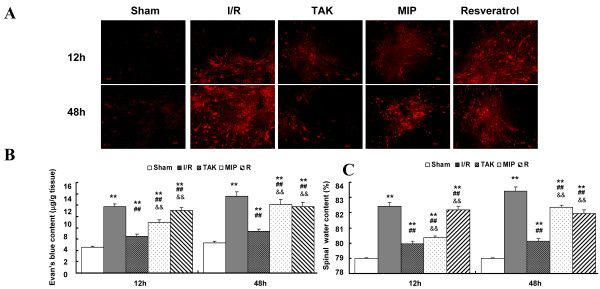
**Effects of TLR4 signaling on BSCB dysfunction after I/R injury. (A)** Representative EB dye after intrathecal injection with TAK-242, MIP, and Resveratrol. Almost no red fluorescence was seen in spinal cord parenchyma in the Sham group at 12 and 48 h after injury. Much more red fluorescence could be seen in the I/R and Resveratrol groups at 12 h. These increased in intensity at 48 h in all groups after injury, especially in the gray matter of the I/R and MIP groups. Minimal EB red fluorescence was seen in the Sham and TAK groups at the above two time points. **(B)** Quantification data of EB content of spinal cord (μg/g). **(C)** Quantification of the water content of the spinal cord. All data are represented as mean ± SEM (n = 8 per group). Scale bars = 50 μm for 100× images. **P < 0.01 vs. Sham group; ## *P* < 0.01 vs. I/R group; &&*P* < 0.05 vs. TAK group.

We next determined the role of the TLR_4_ signaling cascade in BSCB leakage. As shown in Figure 5A, maximal leakage, as monitored by amount of EB extravasation, was noted in the I/R group at 12 and 48 h after surgery (*P* < 0.01 vs. Sham group). Intrathecal pretreatment with TAK-242 prevented the increased leakage of BSCB at both time points recorded (both *P* < 0.05 vs. I/R group). Comparing EB extravasation among groups, highly intense red fluorescence was observed in the I/R and Resveratrol groups at 12 h, which intensified at 48 h in all groups, especially in the gray matter of the I/R and MIP groups. Minimal EB extravasation was detected in the Sham group at the above two time points (*P* < 0.05 vs. I/R group).

Additionally, assessments of water content showed similar results at 12 and 48 h after surgery, as seen in Figure 4C. It could be inferred that I/R increased the water content due to spinal cord edema (*P* < 0.05 vs. Sham group), while intrathecal transplantation with TAK-242 attenuated the increase in water content (*P* < 0.05 vs. I/R group). Of note, the depletion of MyD_88_ by intrathecal transplantation with MIP only attenuated these effects at 12 h (*P* < 0.05 vs. I/R group); a similar effect was observed at 48 h in the group lacking TRIF function (*P* < 0.05 vs. I/R group).

### Effects of the TLR_4_ pathway on NF-κB activation and inflammatory cytokines after I/R

**Figure 6:**
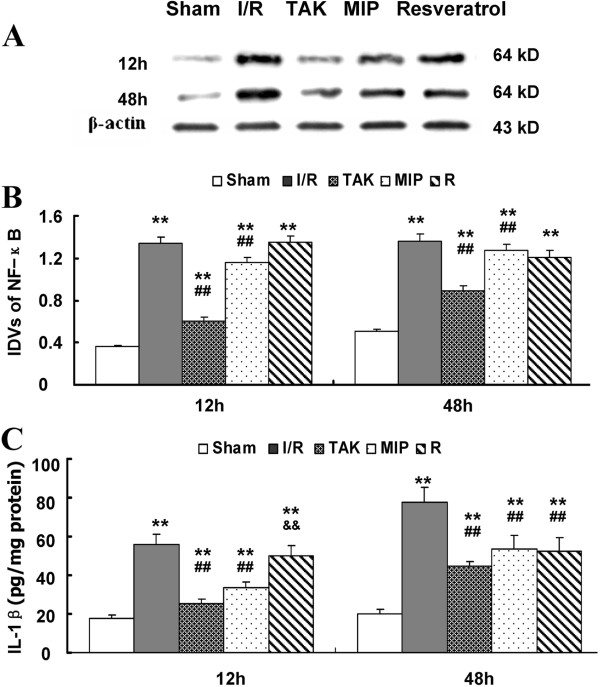
**Effects of TLR**_**4 **_**signaling on NF-κB activation and inflammatory cytokines after I/R. (A)** Representative Western blot and quantitative protein analysis of NF-κB in the spinal cord at 12 and 48 h after intrathecal pretreatment with TAK-242, MIP, and Resveratrol for 3 days. **(B)** The relative integral density values were calculated after normalizing against β-actin in each sample. **(C)** Quantification of IL-1β production in spinal cord at 12 and 48 h after I/R injury, as assessed by ELISA. **P < 0.01 vs. Sham group; ## *P* < 0.01 vs. I/R group; &&*P* < 0.05 vs. MIP group.

Finally, we examined the levels of NF-κB and its downstream inflammatory cytokine proteins after intrathecal pretreatment with TAK-242, MIP, or Resveratrol. As presented in Figure 6, I/R injury greatly induced NF-κB in comparison with the sham group at 12 and 48 h after surgery (*P* < 0.05 vs. Sham group), and such induction was accompanied with significantly increased IL-1β (*P* < 0.05 vs. Sham group). Pretreatment with TAK-242 inhibited NF-κB and IL-1β expression in the spinal cord after injury at both time points (*P* < 0.05 vs. I/R group). To compare the effects on NF-κB and IL-1β after pretreatment with MIP or Resveratrol, NF-κB and IL-1β were detected in the I/R group and the Resveratrol group at 12 h; both NF-κB and IL-1β were significantly increased in all groups at 48 h, especially in the rats of the I/R and MIP groups (*P* < 0.05 vs. I/R group).

## Discussion

Spinal cord I/R injury after thoracic aortic surgery is invariably associated with dysfunction of the BSCB and plays a fundamental role in the progression of several unpredictable and disastrous complications, such as spinal cord swelling and secondary nerve injury, which account for much of the morbidity and mortality of this condition [4,5]. Spinal cord edema is often long lasting and resistant to therapeutic intervention. Recently, TLRs, especially TLR_4_, have gained extensive attention for their important roles in various models of I/R injury [1,18]. In our previous study, we demonstrated that increased activation of TLR_4_ in the spinal cord was associated with increases of BSCB leakage after I/R injury. Our present study is the first attempt to further characterize the inflammation that occurs in a specific cell population after spinal cord I/R injury and suggests the functional relevance of TLR_4_-mediated downstream signal transduction during the bimodal stage after injury.

With an improved understanding of spinal cord I/R injury, the damage sustained during thoracoabdominal aortic occlusion has been classically defined as an initial ischemic event and a delayed reperfusion injury, corresponding with the clinical presentation of bimodal distribution of spinal cord injury [1-3]. Clinically, the manifestation of immediate severe spinal cord injury is rare. Usually, patients experience delayed neurological deficits and develop paraplegia in the several hours to days after surgery, which highlights the pathological mechanisms defined by the responses in the ischemic phase, and an ongoing amplification of molecular insults during the reperfusion phase [23]. After I/R injury, the cytokines released by local cellular populations in the early phase may cause increased accumulation of inflammatory cells, and the chemokines released amplify inflammatory responses, leading to the clinical presentation of functional deficits [24,25]. As demonstrated in our previous study, increased BSCB leakage closely associated with invasion of exogenous ligands inducing immune and inflammatory responses were found during the course of spinal cord I/R injury, as evaluated by extravasation of EB dye [8,9]. Similarly, in this study, the bimodal distribution of EB extravasations was clearly observed as two phases, one stage from 6 to 18 h after surgery which continued for several hours, and the later one began at 36 h and peaked at 48 h (Figure 1), paralleling the clinical manifestation of an early ischemic phase with great risks of delayed and exacerbated injury, such as paralysis, two days after surgery [4,5].

A growing body of evidence indicates the important role of TLR_4_ in the evolution of an inflammatory reaction after I/R injury [16,19]. The stimulation from ischemic or necrotic cells causes TLR_4_ activation, subsequent translocation of NF-κB to the nucleus, and the production of proinflammatory cytokines and chemokines [10,16]. Similarly, the immunohistochemistry data in our study confirm the essential role of TLR_4_ in the inflammatory processes associated with increased BSCB leakage. Significantly, our study lies in the understanding of the correlation between the TLR_4_-mediated MyD_88_-dependent and TRIF-dependent signaling pathways during the bimodal stage after I/R injury. Therefore, we chose 12 and 48 h time points at which maximal changes were reached after I/R injury to further explore the role of TLR_4_-mediated downstream signaling. It is essential to develop potential therapies targeting these points to attenuate and avoid subsequent injury. Some investigations have already reported the effects of TAK-242, MIP, and Resveratrol on the inhibiting functions of TLR_4_ and its downstream adaptor receptors, MyD_88_ and TRIF, which receive the stress stimulation [16,19,20]. In this study, we depleted the function of the TLR_4_ signaling pathway by continuous intrathecal injection with TAK-242, MIP, or Resveratrol at a 12-h interval 3 days before surgery. The effects of the inhibitors were detected by Western blot. Based on our results, the protein levels (Figure 3A-C) and integrated density values (Figure 3D-F) of TLR_4_, MyD_88_, and TRIF in the I/R group were obviously higher than those in rats intrathecally pretreated with TAK-242, MIP, or Resveratrol, as well as those in the Sham group at both observed time points. Therefore, TAK-242, MIP, and Resveratrol employed before reperfusion significantly prevented their functions from receiving the reperfusion-induced stimulation. However, pretreatment with only one of the inhibitors lead to changes in three indicators (TLR_4_, MyD_88_, and TRIF) in each group simultaneously. Therefore, it is important to further assure specificity of each inhibitor by detecting whether there is cross-talk in signal transduction. As tested herein, TAK-242 specifically binds the TLR_4_ receptor without much impact on the functions and expressions of adaptor receptors MyD_88_ and TRIF downstream. Likewise, MIP and Resveratrol were proven to solely decrease the protein expression of MyD_88_ and TRIF, respectively, demonstrating the reliability of intrathecal injection with specific binding proteins to explore the role of the TLR_4_ pathway, as performed in our study (Figure 3).

Our study showed that I/R injury caused vascular events of BSCB in segments most vulnerable to ischemia and exhibited more pronounced vascular disruptions at 48 h than those at 12 h after surgery. The BSCB act as a metabolic barrier to strictly regulate molecular exchange between the circulating blood and spinal cord, and strictly controls the spinal cord microenvironment required for normal neuronal function and micro environmental homeostasis [6,9,26]. Increased BSCB leakage would expose the spinal cord to endogenous ligands and exogenous invading pathogens in the circulation and aggravate inflammatory responses and neurotoxic effects in the development and recovery associated with spinal cord injury, resulting in irreversible damage to the physiological functions of aesthesia and kinesis, as well as to bladder, bowel, and sexual functions following the injured plane. Moreover, the BSCB may determine the innate immune reaction [26]. Therefore, the exploration of the cellular compositions of the BSCB implicated in TLR_4_ activation will greatly contribute to the better understanding the physiological function or pathological development in the early and late phases of inflammation after I/R injury. As reported in previous studies, glial activation occurred in response to ischemia and the responses were very rapid, especially in microglia, which changed in morphology and increased in number within the first few minutes to hours after reperfusion [1,3,7,26], while astrocytes were observed to be mildly increased over 8 h after reperfusion [26]. Similarly, the microglia increased in number and showed intense staining in the spinal cord after surgery and peaked at 12 and 48 h, whereas the GFAP staining became intense 24 h after reperfusion and peaked at 48 h, suggesting that increased BSCB leakage may be attributed to glial activation through their membrane-bound receptor TLR_4_ which is closely associated with inflammatory reactions (Figure 4). Increasing evidence has shown that there is an important and complicated signal transmission within activated neuralgia in response to spinal cord injury [6,7,18]. To explain the different activation phases of glial cells, one can easily consider the different degrees of BSCB leakage and the different roles of glial cells involved. Perivascular microglia surrounding the BSCB are considered as the first actors to remove tissue, cells debris, or macromolecular proteins penetrating through functional leakage of the BSCB, as suggested in the model of spinal cord ischemia and compressive injury [7,26,27]. Astrocytes have been demonstrated to play a major role in homeostasis of the BSCB and recovery of neurological function after ischemia [7]. In the present study, compared with the widespread activation of microglia both in the early (12 h) and late phase (48 h) after surgery, the increases in GFAP immunoreactivity were mainly distributed in spinal gray matter, where the capillaries were more numerous and formed a dense capillary bed in comparison with white matter. Thus, astrocytes were considered to be hyper-responsive states in cases of organic damage of BSCB. In addition, the effects of the inhibitors on double-labeled glial cells were chosen to quantify at 48 h after surgery for the maximal activation based on micrographs. As shown in Figure 4B and C, compared with I/R group, the number of double-labeled microglia and astrocytes in groups pretreated with TAK-242, MIP, and Resveratrol, respectively, decreased the most in the TAK group, suggesting that the relatively intact microenvironment of the spinal cord contributed to the weak responses of glial cells in the BSCB lacking the functional TLR_4_ receptor after surgery. Comparing the effects of MIP and Resveratrol treatment, immunofluorescence staining and quantification data demonstrated that I/R-induced elevated double-labeled microglia and astrocytes were significantly decreased in the Resveratrol group, whereas less so in the MIP group, indicating a major involvement of the TLR_4_-TRIF-dependent pathway 48 h after surgery.

Up regulating TLR4 and NF-κB was accompanied with the production of proinflammatory cytokines. As expected, our results revealed increases in NF-κB and IL-1β with increasing time after I/R injury, signifying the amplified and aggravated inflammatory responses in pathogenesis in the spinal cord. Previous studies have demonstrated the capacity of TLR_3_ to activate TRIF [15,28] and that TLR_4_ directly activated MyD_88_[16,19]. With recent advancements in this area, some studies have indicated that TLR_3_is not indispensable for the TRIF-dependent pathway while the TLR_4_ receptor has the potential to activate the TRIF-dependent pathway upon receiving a signal from MyD_88_-dependent pathways [15]. The signals that bridged through TICAM-_2_ to TICAM-_1_ led to the recruitment of TRIF and TRAM in a cascade [15,29], resulting in the rapid production of cytokines and the late phase activation of NF-κB and mitogen-activated protein kinases, thus exerting positive feedback on MyD_88_-activation [30,31]. Therefore, based on our data, the ongoing cascade of inflammatory responses involves the activation of both the MyD_88_ and TRIF pathways.

IL-1β is the most commonly implicated protein in inflammatory responses and exhibits biphasic distribution in various models of I/R injury [2,3,32]. These studies revealed that IL-1β significantly increased and remained high in the reperfusion period, and that heightened IL-1β were associated with both early ischemic and late reperfusion injury [2,3,32,33]. Based on our results, we found analogously biphasic IL-1β responses during the reperfusion period since the levels expressed at 12 and 48 h were much higher than those at 24 h and were in accordance with the activation of NF-κB. It was intriguing to explore the temporal relationship between TLR_4_/MyD_88_ and TLR_4_/TRIF. Similar to the results in our study, O’Neill and Bowie suggested that the expression of NF-κB occurred in two phases after TLR_4_ activation [17]: MyD88 mediated events in the early phase, while TRIF mediated the later phase. We found that there was a significant increase in NF-κB release and IL-1β production at 12 h. Nevertheless, contrasting results were observed at 48 h in the Resveratrol group (Figure 4), indicating that MyD_88_ and TRIF dependence occurred in the early and late phases, respectively.

Of note, sequential activation of microglia and astrocytes were supported by immunofluorescence staining, but not necessarily in that order given the complexity of *in vivo* experiments. Nevertheless, a sustained majority of TLR_4_ expression occurs in microglia throughout the inflammatory responses, and the increasing involvement of astrocytes with time in the late phase might be an explanation for the delayed neurological deficits [3,7,26,27,34]. Further *in vitro* studies still need to be conducted to identify the development and mode of action of these inflammatory mediators to better elucidate the mechanism underlying the pathogenesis.

Taken together, our data suggest that there could be different phases of spinal cord I/R injury. Inflammation is a subsequent event during the bimodal stage after injury. The early phase of I/R injury in the spinal cord was found to be largely TLR_4_/MyD_88_-dependent and microglia-dependent, and the following late phase was found to be mainly dependent on TLR_4_/TRIF activation, which was amplified by MyD_88_ signaling with the involvement of both microglia and astrocytes. These findings explain why several therapeutic treatments have failed in patients with I/R injury. A novel and ideal therapy that would address the different phases of injury and inflammation may be valuable in preventing delayed injury and its clinical manifestations.

## Abbreviations

BSCB: Blood-spinal cord barrier; CD13: Aminopeptidase-N; CD31: Platelet endothelial cell adhesion molecule-1; EB: Evan’s Blue; GFAP: Glial Fibrillary acidic protein; Iba-1: Ionized calcium-binding adaptor molecule 1; IL: Interleukin; I/R: Ischemia-reperfusion; MIP: MyD_88_ inhibitory peptide; MyD88: Myeloid differentiation factor_88_; NF-κB: Nuclear factor kappa-B; TLR4: Toll-like receptor_4_; TRIF: TIR domain-containing adaptor-inducing IFN-β.

## Competing interests

The authors declare that they have no competing interests.

## Authors’ contributions

X-QL and BF participated in the animals’ care and made all the animal models. X-QL, HW and W-FT participated in tissue preparation, and sectioning and performed most immunohistochemistry; X-QL, BF and W-FT performed Western blotting assay and statistical analysis; HM involved in the guide of model design and study design; H-WL gave important directions to data analysis and manuscript writing. All authors read and approved the final manuscript.
